# Adverse and benevolent childhood experiences among adults in the United Kingdom: a latent class analysis

**DOI:** 10.1186/s12889-024-19448-z

**Published:** 2024-07-30

**Authors:** Shannon M. Cain, Emily A. Rooney, Samantha Cacace, Abigail Post, Kirsten Russell, Susan Rasmussen, Justin C. Baker, Robert J. Cramer

**Affiliations:** 1 Department of Epidemiology and Community Health, University of North Carolina at Charlotte, 9201 University City Blvd., Charlotte, NC 28227 USA; 2 Violence Prevention Center, University of North Carolina at Charlotte, 9201 University City Blvd., Charlotte, NC 28227 USA; 3 Department of Psychiatry and Behavioral Health, The Ohio State University Wexner Medical Center, 1670 Upham Drive 1st Floor, Columbus, OH 43210 USA; 4 Department of Psychological Sciences and Health, University of Strathclyde, 40 George Street, Glasgow, G11QE UK

**Keywords:** Adverse childhood experiences (ACEs), Benevolent childhood experiences (BCEs), Psychological distress, Suicide, Latent class analysis

## Abstract

**Background:**

Adverse childhood experiences (ACEs) are important factors for population mental and physical health. While considerable public health literature demonstrates the global relevance of ACEs, more recent research shows that benevolent childhood experiences (BCEs) might be important to consider in their direct and mitigating roles for psychological distress and other mental health outcomes. There is little evidence of latent class examinations involving both ACEs and BCEs among adults in western nations. The present study sought to replicate and extend prior literature by: (1) assessing the extent to which past latent class groupings reproduce in present samples, and (2) analyzing the association of latent classes of childhood experiences with psychological distress and suicidal thoughts and behaviours (STBs). We examined psychological distress (i.e., depression, anxiety, post-traumatic stress, general wellbeing) and STBs (i.e., suicidal ideation, self-harm ideation and behaviour, entrapment, and defeat).

**Method:**

Data were drawn from two nationwide cross-sectional online survey studies in the United Kingdom. The first sample (*N* = 488) was drawn from a study on suicidal behaviour, and the second sample (*N* = 447) was from a study concerning risk for interpersonal violence.

**Results:**

Results largely replicated an existing four class solution of childhood experiences: Class 1 (*Moderate ACEs/High BCEs*; 17.6%), Class 2 (*High ACEs/Moderate BCEs*; 15.3%), Class 3 (*Low ACEs/High BCEs*; 48.3%), and Class 4 (*Low ACEs/Moderate BCEs*; 18.8%). Class 2 (*High ACEs/Moderate BCEs*) was associated with consistently worse psychological distress and STBs. Classes containing high BCEs (1 and 3) were characterized by generally lower levels of psychological distress and STBs.

**Conclusions:**

Results affirm the potential value for jointly considering ACEs and BCEs to understand psychological distress and STBs. ACEs and BCEs may serve foundational roles in theories of suicide. The protective role of BCEs hypothesized in resiliency theory may be supported. Prevention practice and research implications are discussed.

**Supplementary Information:**

The online version contains supplementary material available at 10.1186/s12889-024-19448-z.

## Background

Early childhood experiences have a significant impact on health and wellbeing throughout the life course. In a landmark study conducted by Felitti and colleagues [1], the concept of adverse childhood experiences (ACEs), understood as negative psychosocial events that occur early in life, emerged as an important indicator of health. Specifically, this research identified 10 harmful life experiences related to physical, sexual, and emotional abuse, parental/caregiver neglect, and household/community disfunction during childhood and adolescence (ages 0–17; [1, 2]). The ACE scale was developed and validated to assess these 10 experiences [1]. ACEs are a major public health problem, as it has been estimated between 50 and 71% of United Kingdom (UK) adults have experienced at least one ACE before age 18 [3–6]. A higher number of ACEs were also associated with higher levels of perceived stress and worse psychological distress in adults [7]. Since then, these findings have been validated in several studies spanning Africa, Asia, Australia, Europe, and the United States [8]. As such, the ACE Scale has been used to measure these experiences in relation to childhood trauma and health across the globe [2, 3].

Recently, benevolent childhood experiences (BCEs), the counterpart to ACEs, received attention in the literature. BCEs are positive early life experiences that are theorized to serve as a buffering impact against ACEs, subsequent psychological distress and other negative outcomes [9]. Research on BCEs has expanded since the development of the BCEs Scale [10]. Since its development, several validation studies examined the BCEs Scale among community samples of adults in China [11] and Portugal [12], Turkish students [13], and parents experiencing housing difficulties in the United States [14]. The BCEs scale examines positive experiences centered around safety, security, and comfort. For instance, the scale assesses the presence of interpersonal relationships (e.g., friends, caregivers) and necessities such as a home routine (i.e., regular meals and bedtime). Higher levels of BCEs are predictive of lower psychological distress [10].

Although studies primarily examined ACEs and associated outcomes, BCEs are also crucial to investigate, as they may serve as protective factors against stress and psychological distress [15]. Further, there is evidence showing BCEs and ACEs exist on separate continuums as distinct constructs. For example, in a study examining the latent factor structure of the ACEs and BCEs scales, results supported a two-factor model (i.e., ACEs and BCEs subscales that were negatively correlated) that demonstrated a significantly better fit than a one-factor model [16].

Understanding ACEs and BCEs as distinct, yet related, constructs is important for empirical research, screening, public health surveillance, and clinical practice. The present study contributed to the integrated study of ACEs and BCEs in two ways. First, we conducted one of the first latent class analyses to assess possible childhood experience subgrouping replication across countries. Doing so may inform theory development, classification of childhood experiences in public health surveys, and clinical assessment of childhood experiences when working with adult community members. Second, we assessed latent classes of childhood experiences across several outcomes concerning psychological distress (e.g., symptoms of depression and anxiety) and suicidal thoughts and behaviours (STBs). Observed variation in psychological distress and STBs may inform risk assessment and therapeutic treatment selection for persons experiencing these clinical matters.

## What do we know about ACEs, BCEs, and wellbeing?

ACEs are important to assess as the potential consequences of exposure to adverse experiences are well-documented. ACEs have been associated with poor physical health outcomes (e.g., obesity; [17]) and poor social-behavioural consequences (e.g., aggressive behaviour; [18, 19]). ACEs have also been associated with symptoms of psychological distress, including anxiety, depression, and post-traumatic stress, as well as STBs [1, 20, 21]. Furthermore, research has consistently demonstrated that experiencing more ACEs is associated with an increased risk of worse physical and mental health outcomes in adulthood [22, 23].

While the study of ACEs gives a necessary spotlight to the effect of childhood trauma on adult health (e.g., [24]), the study of BCEs helps complete this picture by examining the influence of positive childhood experiences on adult outcomes. Although the exact prevalence of BCEs in the UK is not fully understood, recent research suggested up to 95% of UK adults have experienced multiple BCEs [25]. Moreover, literature suggests that BCEs promote and protect the health of individuals. For instance, one study found higher levels of BCEs were associated with effective stress management, and good physical and dental health [9]. Other studies suggested that BCEs are negatively associated with stress and anxiety [26], poor cardiovascular health [27], and sleep disorders [28].

## What do we know about latent classes of childhood experiences?

The current literature examining ACEs and BCEs together demonstrates higher levels of ACEs are associated with higher psychological distress and, conversely, higher levels of BCEs are associated with less psychological distress. For instance, Doom and colleagues found higher ACEs were associated with higher levels of depression in a sample of undergraduate students. They also found higher levels of BCEs were associated with lower levels of psychological distress (i.e., depression, perceived stress, loneliness) [15].

When types of childhood experiences are considered together, there are several hypothesized mechanisms explaining the impact of both ACEs and BCEs on psychological distress and STBs. A widely held view suggests toxic stress is induced by experiencing multiple, chronic ACEs. In turn, toxic stress may negatively affect processes such as gene expression and cognitive and emotional development [29]. Resiliency Theory [30] has been used to frame the understanding of the potential interaction between ACEs and BCEs. Consistent with interpersonal and ecological viewpoints, Resiliency Theory posits that resilience develops over time and within differing contexts, affected by the intersection of multiple systems relevant to childhood (e.g., schools, family unit). Reflecting a characteristic to adapt or overcome, resilience can operate in three distinct ways in the context of ACEs and BCEs [30, 31]. BCEs are thought to have both direct and moderating links with negative health outcomes [31]. For instance, greater BCEs serve as a direct protective factor. Also, greater BCEs may mitigate the effects of ACES on outcomes like psychological distress. The third possibility grounded in Resiliency Theory is that experiences of moderate ACEs alone facilitate development of resilience to future trauma and distress. Recent evidence supports the possible stress-buffering role of BCEs (i.e., the second mechanism posited by Resiliency Theory; [31]), as the presence of a high number of these childhood experiences in combination with high ACEs was associated with lower odds of stress and depression in a sample of Chinese undergraduates [32].

Studies comparing ACEs and BCEs primarily used regression analysis to determine significant associations. This straightforward approach to examining ACEs and BCEs is limited in that it treats both types as truly independent constructs. Another view of childhood experiences is that varying sub-groupings may yield differing levels of wellbeing. Latent class analysis (LCA) can facilitate such a nuanced examination. Only two known studies to date conducted LCAs using ACEs and BCEs [33, 34]. LCA is a statistical procedure used to detect latent heterogeneity in samples [35]. Subgroups are identified based on patterns of individuals’ responses to observed variables.

The two studies using LCA to examine ACEs and BCEs used responses to individual scale items to determine class membership. In the first study, Johnson and colleagues examined ACEs and BCEs conjointly in a single LCA model among subgroups of parents from the UK, US, Canada, and Australia. Using items from the ACEs and BCEs Scales, they identified four latent classes: (1) low-ACEs/high-BCEs; (2) moderate-ACEs/high-BCEs; (3) moderate-ACEs/low-BCEs; and (4) high-ACEs/moderate-BCEs [33]. They also found there was an increased risk of parent and child psychological distress and family dysfunction among those reporting moderate-to-high levels of ACEs, regardless of the level of BCEs reported. Their findings highlight that the risks associated with exposure to adversity may be difficult to mitigate even with the presence of positive experiences. In the second study, Tang and colleagues examined patterns of ACEs and BCEs, but conducted separate LCAs for each scale in a Chinese student sample [34]. Using items from the ACEs and BCEs Scale, their results revealed three latent classes for ACEs: (1) emotional abuse; (2) high ACEs; and (3) low ACEs. They also found four latent classes for BCEs: (1) relationship support; (2) low BCEs; (3) high BCEs; and (4) high quality of life. Individuals with emotional abuse and high ACEs class had an increased risk of more severe depressive symptoms and suicidal ideation, whereas a high quality of life and high BCEs served as protective factors for psychological distress.

## The present study

ACEs and BCEs are widely relevant to public health surveillance (e.g., [36]), and research (e.g., [25]) for community dwelling adults. Likewise, childhood experiences can inform screening, assessment and treatment of psychological distress and suicide [37]. As such, the present study extended existing LCA work on childhood experiences in two samples of UK-based adults. Two types of outcomes were assessed: psychological distress (i.e., symptoms of depression, anxiety, post-traumatic stress, and mental wellbeing) and STBs (i.e., entrapment, defeat, suicidal thinking, and self-harm ideation and behaviour). The present study examined the following aims and hypotheses (H).

Aim 1: To assess the extent to which LCA groupings replicate in present samples.

H1: We hypothesized that a 4-class model will best fit the ACEs/BCEs data.

Aim 2: To analyze the association of latent classes of childhood experiences with psychological distress and STBs.

H2: We hypothesized classes characterized by high ACEs and low BCEs will be associated with higher psychological distress and STBs.

For LCAs we drew from both study samples for the initial class testing (Aim 1) and descriptive analyses by psychological distress/STBs (Aim 2). Since we drew participants for each aim from across samples, we describe study samples in order, followed by a full results and discussion section.

### Study sample 1: the self-directed violence inflection points study

#### Design

Study sample 1 was drawn from a nationwide online cross-sectional UK survey study centered on narratives of halting suicide and self-injury.

### Procedure^1^

This study was approved by the University of Strathclyde Ethics Committee (#UEC20/70). Study data were collected between 2020 and 2022. The study was advertised on social media (i.e., Twitter, Facebook, LinkedIn, Instagram). Participants were also recruited through the lead university’s student participant pool where students received course credits for taking part in research. The advertisement labeled the study as a survey about improving understanding of factors that contribute to, or protect people from, psychological distress including STBs.

The survey was hosted on Qualtrics; potential participants visiting the Qualtrics link provided in study advertisements were first directed to the participant information sheet (PIS). The PIS described the study, the inclusion criteria (aged 18 years or older irrespective of prior experiences of STBs), its purpose and included information about the confidential and anonymous nature of the study, what types of questions to expect and the expected time to complete the survey (approx. 30 min), mental health resources, contact information for the primary researchers. To provide informed consent, participants checked a box on a separate sheet. All participants provided informed consent to participate in the study. The survey first presented the demographic questions followed by the remaining measures which were not randomized. The measures were not randomized due to ethics board feedback which requested that we ensure the survey never finished on questions which specifically asked about STBs. Following completion, participants were provided with a downloadable debriefing sheet which included information about available mental health resources and the contact details for the researchers. No payments or incentives were provided for participation.

### Measures

**Demographics**. We included questions on the following demographic variables: Age, race, country of birth, relationship status, gender, and sexual identity. Participants self-identified their race and country of birth through open text boxes; these variables were subsequently collapsed into categories (see Table 1). Sexual orientation, gender, and relationship status were all collected via a pre-determined checklist where participants could indicate more than one identity. Gender and sexual identity also contained the option to indicate “other” with a text box to self-label.

**Table 1:** Sample 1 descriptive information

Variable	M (SD)	*n* (%)
Age	30.48 (12.77)	-
Suicidal ideation	6.22 (9.43)	-
Depressive symptoms	2.02 (1.78)	-
Anxiety symptoms	7.93 (5.69)	-
Post-traumatic stress symptoms	4.89 (2.47)	-
*Gender*		
Cisgender man	-	91 (18.7)
Cisgender woman	-	393 (80.5)
Trans or gender diverse (e.g., gender queer)	-	4 (0.8)
*Sexual orientation*		
Bisexual	-	38 (7.8)
Gay	-	11 (2.3)
Lesbian	-	10 (2.0)
Heterosexual	-	412 (84.4)
Pansexual	-	4 (0.8)
Asexual	-	1 (0.2)
Queer	-	1 (0.2)
Multiple identities	-	9 (1.8)
Other sexual minority	-	2 (0.1)
*Race*		
White	-	435 (89.1)
Multiracial	-	9 (1.8)
United Kingdom nation indicated	-	37 (7.6)
Asian	-	4 (0.8)
Other	-	1 (0.2)
Missing	-	2 (0.4)
*Relationship status*		
Single, not dating	-	140 (28.7)
Casually dating	-	36 (7.4)
In a committed relationship with one person	-	312 (63.9)
*Geographic region of birth*		
United Kingdom	-	456 (93.4)
Africa	-	5 (1.0)
Continental Europe	-	16 (3.3)
North America	-	5 (1.0)
Other (not indicated)	-	5 (1.0)
Missing	-	1 (0.2)

Notes: *N* = 488; M = Mean; SD = Standard deviation

**Negative childhood experiences**. A 10-item version of the Adverse Childhood Experiences Questionnaire (ACE; [1]) was used to establish exposure to negative life experiences during the first 18 years of life. The ACEs measure assesses the presence or absence of the following negative experiences; emotional or physical abuse, sexual abuse, and physical and emotional neglect as well as the individual’s exposure to maternal abuse, parental separation, and/or a household member’s substance abuse, mental illness, or incarceration. Questions are scored as yes (1) or no (0) which allows a cumulative score of ACEs to be generated. Estimated internal reliability in the current sample was acceptable (*KR20* = 0.68).

**Positive childhood experiences**. The Benevolent Childhood Experiences Scale (BCE; [10]), was used to assess positive childhood experiences in the first 18 years of life. The scale consists of 10 items which can be answered by yes (1) or no (0). Items pertain to perceived safety and support (e.g., “at least one safe caregiver”, “at least one good friend”) and internal and external motivation (e.g., beliefs that gave comfort, enjoyment of school, a teacher who cared). A cumulative score is generated ranging from 0 to 10. Higher number of questions answered with “yes” indicates higher levels of positive childhood experiences. The scale has demonstrated adequate psychometric properties [10, 12]. Estimated internal reliability in the current sample was acceptable (*KR20* = 0.71).

**Depression**. To capture experiences of depressive symptoms, we included the Patient Health Questionnaire-2 (PHQ-2; [38]). The instrument possesses high internal consistency, with robust sensitivity and specificity [39]. Each item is rated on a 4-point (0–3) scale with a higher score indicating higher depressive symptomatology. A cut-score of 3 on the total score is considered ideal for a positive screen for possible depression [38]. Estimated internal reliability in the current sample was acceptable (ordinal α = 0.92).

**Anxiety.** We included the Generalized Anxiety Disorder-7 (GAD-7; [40]) as a measure of symptoms of generalized anxiety disorder. Each item is rated on a 4-point (0–3) response scale. The measure possesses high internal consistency, acceptable sensitivity and specificity, and construct validation with measures of wellbeing [41]. Cut-off scores can be derived to differentiate minimal, mild, moderate, and severe levels of anxiety symptoms, with a total score of 5 or higher indicating non-minimal levels of anxiety [40]. Estimated internal reliability in the current sample was acceptable (ordinal α = 0.94).

**Post-traumatic stress symptoms**. The Posttraumatic Checklist-2 (PCL-2; [42, 43]) was included as a brief screener of stress symptoms. The measure includes two items which pertain to intrusive memories and distress associated with reminders of the traumatic event. Each item is rated on a 5-point (1–5) scale and suggests a cut-off score of 4 on the total score be used as an indication of a positive screen for probable posttraumatic stress concerns. The PCL-2 has been found to have good psychometric properties and potential utility as screening instruments [44]. Estimated internal reliability in the current sample was acceptable (Cronbach’s α = 0.87).

**Suicidal ideation**. Suicidal ideation was measured using the Suicidal Ideation Attributes Scale (SIDAS; [45]). The SIDAS includes five items to assess the frequency, controllability, attempt likelihood, level of distress associated with suicidal thinking, and impact on a person’s daily functioning. Responses to the questions are captured using a scale which ranges from 0 (e.g., never) to 10 (e.g., always) (Range 0–50) with a higher score indicating more severe suicidal thoughts. The scale has been reported to have a high internal consistency, good test-retest reliability as well as convergent and discriminant validity [46]. Estimated internal reliability in the current sample was acceptable (Cronbach’s α = 0.75).

### Study & sample 2: the United Kingdom hate-motivated behaviour survey

#### Design

Study sample 2 was drawn from a nationwide UK online cross-sectional survey study investigating experiences of bias-motivated behaviour.

### Procedure^2^

This investigation adhered to the British Psychological Society’s ethical guidelines for internet-mediated research and approval was obtained from the University of Strathclyde’s Ethics Committee prior to commencing data collection (#UEC21/83). The investigation was conducted online via Qualtrics. Data collection took place over a five-month period (December 2022-April 2023). The study was advertised on online platforms (Twitter and Facebook), as well as through a university research recruitment platform. Posters advertising the research were also placed around the campus of one university in Scotland and included a QR code to access the survey. The advertisement labeled the study as a survey about factors that might make individuals more or less likely to become involved in interpersonal violence. All individuals were informed that, to be eligible to participate in the study, that they had to be over the age of 18 and currently living in Scotland.

Participants were given access to a detailed information sheet, outlining the nature and duration of the study, aforementioned inclusion criteria, contact details of the researchers, and information regarding relevant mental health and victim support organisations. The participant information sheet also reinforced the anonymous and confidential nature of the study and provided further information regarding the types of questions that participants would be invited to complete within the survey. Informed consent was requested before participants accessed the survey. All participants provided informed consent to participate in the study. After providing consent, participants completed a basic demographics questionnaire, followed by a range of measures which were presented in a randomized order. The survey took approximately 30 min on average to complete. Once participants had completed the survey, they were provided with a downloadable debrief sheet which restated the purpose of the study, provided contact details for researchers and highlighted local mental health and victim support organisations. Participants who were recruited through the university research recruitment platform received course credits for participation. No other payments or incentives were provided.

### Measures

**Demographics**. We included questions on the following demographic variables: age, gender, sexual orientation, race/ethnicity, and national identity. Gender identity was captured using the following options: male, female, non-binary, male-to-female, female-to-male, queer, prefer not to say, and option not listed. Other than age (reported in years), demographics were collected via a series of checklists (see Table 2). All demographic response options included a choice of “not listed” where participants were invited to provide a text response.

**Table 2:** Sample 2 descriptive information

Variable	M (SD)	*n* (%)
Age	22.84 (7.27)	-
Estimated annual income	£13,346.98 (£12,620.58)	-
Total adverse childhood experiences	2.17 (2.30)	-
Total positive childhood experiences	8.08 (1.98)	-
Depressive symptoms	5.55 (3.43)	-
Anxiety symptoms	10.74 (4.25)	-
Mental wellbeing	22.14 (5.01)	-
Defeat	23.18 (13.37)	-
Internal entrapment	4.61 (5.57)	-
External entrapment	10.07 (10.17)	-
*Gender*		
Cisgender man	-	76 (17.0)
Cisgender woman	-	353 (79.0)
Trans woman	-	4 (0.9)
Trans man	-	2 (0.4)
Non-binary/agender	-	9 (2.0)
Queer	-	3 (0.7)
*National identity*		
Scottish	-	372 (83.2)
English	-	33 (7.4)
Northern Irish	-	8 (1.8)
British	-	3 (0.7)
Irish	-	4 (0.9)
Malaysian	-	4 (0.9)
Other (e.g., Omani)	-	21 (4.7)
Missing	-	1 (0.2)
*Ethnicity/race*		
White Scottish	-	355 (79.4)
White Irish	-	8 (1.8)
White other British	-	29 (6.5)
White Polish	-	2 (0.4)
Asian/Asian Scottish/Asian British	-	21 (4.7)
African/African Scottish/African British	-	2 (0.4)
Biracial	-	3 (0.7)
Multiracial	-	7 (1.6)
Other White (e.g., Austrian)	-	12 (2.7)
Other	-	6 (1.3)
Missing	-	2 (0.4)
*Sexual orientation*	-	
Bisexual	-	64 (14.3)
Gay	-	10 (2.2)
Lesbian	-	16 (3.6)
Heterosexual	-	300 (67.1)
Queer	-	15 (3.4)
Questioning	-	6 (1.3)
Pansexual	-	13 (2.9)
Asexual	-	3 (0.7)
Prefer no label	-	19 (4.3)
Missing	-	1 (0.2)
*Lifetime self-injury ideation*		
Never	-	226 (50.6)
Ideation in the past	-	170 (38.0)
Current/recent ideation	-	51 (11.4)
*Lifetime self-injurious behaviour*		
Never	-	294 (65.8)
Past behaviour	-	142 (31.8)
Current/recent behaviour	-	11 (2.5)

Notes: *N* = 447; M = Mean; SD = Standard deviation

**Adverse childhood experiences**. We used the same 10-item version of the Adverse Childhood Experiences Questionnaire (ACE Questionnaire; [1]) as in study sample 1. Estimated internal reliability in the current sample was acceptable (*KR20* = 0.79).

**Positive childhood experiences**. We again used the same Benevolent Childhood Experiences Scale (BCE; [10]) used in the first study. Estimated internal reliability in the current sample was acceptable (*KR20* = 0.72).

**Depressive and anxious symptomology**. The Hospital Anxiety and Depression scale (HADS) contains two 7-item subscales that assess depressive and anxious symptomology respectively. Participants are asked to indicate the extent to which they have experienced symptoms in the past week on a Likert scale ranging from 0 to 3 and higher item scores indicate higher depression symptomatology [47]. Total scores for each subscale are calculated by taking a sum of responses. The HADS is a valid and reliable measure, which is frequently used in community settings [48, 49]. Both subscales have been found to be internally consistent (anxiety: α = 0.80 and depression: α = 0.76) [50]. In the current sample, internal estimated reliability of the depression subscale (ordinal α = 0.87) and anxiety subscale (ordinal α = 0.74) were both acceptable.

**Positive mental well-being**. The short version Warwick-Edinburgh Mental Wellbeing Scale (SWEMWBS) comprises 7 positively worded items that relate to different aspects of positive mental health [51]. The scale has five response categories ranging from 1 (“None of the time) to 5 (“All of the time”). Higher scores indicate more positive mental wellbeing. The measure has been shown to have good internal consistency (α = 0.91; [51]) and has been validated among the Scottish adult population [52]. Estimated internal reliability in the current sample was acceptable (Cronbach’s α = 0.88).

**Self-injury ideation**. Thoughts of self-injury were assessed using a single-item measure. Participants were asked if they had ever seriously thought about taking an overdose (e.g. of pills or any other medication) or trying to harm themselves (e.g. by cutting) but not actually done so. The response options presented to the participants were: Never (0), I have done so in the past but not anymore (1), or I currently have these thoughts (2). This measure has been used in previous community-based investigations of self-harm [53, 54].

**Self-injurious behaviour**. Acts of self-injury were assessed using a single-item measure. Participants were asked if they had ever deliberately taken an overdose (e.g. of pills or other medication) or tried to harm themselves in some way (e.g. cutting themselves). Similar to the item implemented to assess self-harm thoughts, participants could select the following options: Never (0), I have done so in the past but not anymore (1), or I currently harm myself (2). This measure has been used in previous work conducted in community samples [54, 55].

**Defeat**. The Defeat Scale is a 16-item measure that assesses an individual’s feelings of defeat (i.e., perceived failed struggle and loss of social rank). Respondents indicate the occurrence of these perceptions on a 5-point scale ranging “Never” (0) to “Always” (4) [56]. Three items (2, 4, and 9) are reverse coded prior to calculating the total score. Scores for each item are combined to create a total continuous score with higher scores indicating greater levels of defeat. The measure has been widely used, has demonstrated excellent internal consistency (α = 0.96; [57]) and has demonstrated concurrent validity with other measures of social rank [58]. Estimated internal reliability in the current sample was acceptable (Cronbach’s α = 0.95).

**Entrapment**. Perceptions of being trapped were assessed using the 16-item Entrapment Scale [56]. The measure consists of two subscales: internal entrapment (perceptions of entrapment by one’s own thoughts and feelings; 6 items) and external entrapment (perceptions of entrapment by external situations; 10 items). Both subscales were implemented in the current study. Respondents rate the extent to which each item describes their feelings on a five-point scale that ranges from 0 (“Not at all like me”) to 4 (“Extremely like me”). Responses to each item are combined to create a total score for each subscale and higher scores indicate higher feelings of entrapment. The Entrapment Scale has been demonstrated to have good test-retest reliability [59] and excellent internal consistency (α = 0.86–0.94) [56]. Estimated internal reliability in the current sample was acceptable (Cronbach’s α = 0.96).

### Analytic plan

Data preparation for each study took place using SPSS v. 26. The online supplement contains details for data preparation and quality checks for each study. To assess Aim 1, using Stata v. 18 [60], responses from the ACEs and BCEs were randomly sampled from the Inflection Points study and hate-motivated behaviour (HMB) study for a total of 400 participants (*n*_*inflection*_ = 218; *n*_HMB_*=* 182). This sampling strategy was used for the initial LCA. Participants were included from both samples in the class enumeration step to reduce the likelihood that classes are non-generalizable based on coming strictly from one sample [61]. We used a multivariate non-normal mixture model (MNNMM) to fit 1, 2, 3, and 4 classes using ACEs and BCEs responses. The MNNMM uses a Bernoulli distribution to account for binary, categorical response categories for class enumeration [62].

To assess Aim 2, psychological distress and STB outcomes, namely sum totals for the PHQ2, GAD-7, PCL-2, and SIDAS, were assessed using the remaining 270 participants in the Inflection Points study sample. Since all outcomes for the Inflection Points study sample were continuous, analysis was conducted using the modified Bolck, Croon, and Haganeers 3-step approach [63, 64] to mitigate class drift (i.e., the tendency for individual class membership to change across models with the addition of distal outcomes; see [63]). The Bolck and colleagues’ 3-step approach involves three analytic steps: (1) examination of the measurement model comprising LCA-derived classes; (2) predicted scores are derived from a combination of latent variable parameter estimates (i.e., latent classes) and observed indicator scores (i.e., observed data), and; (3) predicted scores are used as if they are fixed observed indicators to examine distal outcomes, and model fit is evaluated [63]. For the HMB study sample, outcomes, including HADS, WEMWBS, self-harm ideation, self-harm behaviour, defeat, and entrapment, were assessed using the remaining 265 participants. For the HMB sample, we used a manual Vermunt 3-step approach [64] to account for the mixture of binary and continuous outcomes; this approach is similar to the Bolck, et al. procedure, but without continuous distributional assumptions of distal outcomes, given the combined binary and continuous nature of outcomes in the HMB sample. All analyses were conducted using Mplus v. 8.10. [65]. We used the remaining random participants across samples because it is common practice to draw separate samples from collected data to: (a) determine the ideal number of classes for latent class analysis, and; (b) identify distal outcomes predicted by such classes, as we’ve done in the current study. Separating these samples provides confidence that the number of classes identified is a generalizable solution, avoiding mistaken conclusions about class fit based only in one sample or analysis [61]. Using the same sample for determining both the ideal number of classes and distal outcomes analysis significantly reduces statistical power and creates a high chance that class solution fit is merely a function of one dataset, resulting in poor external validity [66].

## Results

### Participants

**Study Sample 1.** Sample demographic, childhood experience, and psychological distress information is contained in Table 1. The average age was in the young-to-middle adult range (M = 30.48, SD = 12.77). The sample was heavily weighted toward cisgender woman gender (80.5%), heterosexual sexual orientation (84.4%), white race (89.1%), and persons currently born in the UK (93.4%). Nearly two-thirds of the sample was in a committed relationship with one person.

**Study Sample 2.** Participant demographics, childhood experiences, and psychological distress information is summarized in Table 2. The sample was of young adult average age (M = 22.84, SD = 7.27) and annual income level was low. The following primary demographic classifications included: cisgender woman (79.0%), Scottish national identity (83.2%), and white Scottish ethnicity/race (79.4%). Diversity was evident with respect to sexual orientation (e.g., approximately one-third of the sample indicated being of sexual minority status). Almost half of the sample reported lifetime self-injurious ideation, and more than one-third indicated lifetime self-injurious behaviour. The mean number of adverse childhood experiences was relatively low (approximately 2 of 10), whereas positive childhood experience average was high (approximately 8 of 10). Depressive symptoms were in the normal range, whereas anxiety symptoms were in between borderline and clinical ranges [47]. Overall mental well-being was in line with national norms established among a sample of adults living in the UK [67]. Defeat, internal entrapment, and external entrapment were all somewhat lower compared to a prior non-clinical sample of UK-based adults [68].

**Aim 1: To assess the extent to which LCA groupings replicate in present samples.** Comparing omnibus fit indices, the highest level of agreement in clustering criteria suggested a 2-class model, while parsimony criteria favored a 4-class model (see Table 3). The 2-class model suggested that 66.9% of the sample would fall into Class 1, for which estimated responses consistently indicated low ACEs scores and high BCEs scores. About 33.1% of the sample would be classified into Class 2, suggesting estimated response probabilities consistently high in ACEs and low in BCEs. Further, classification accuracy was high in the 2-class model, with a 96.5% probability of being correctly classified into Class 1, and 97.3% probability of being correctly classified into Class 2 (see online Supplement Table 1 for 2-class model response probabilities). We examined both a 5-class and 6-class solution in the initial LCA. Both solutions had cases with standard errors exceeding possible thresholds (i.e., incalculable standard errors and poor evidence for stable convergence), and high indication of instability in the model (i.e., where accurate classification probabilities were less than 90%). Also, in the 6-class solution, there was non-positive-definite first-order derivative products in the variance-covariance matrix, indicating instability and possible model non-identification. Collectively, these results indicate both the 5-class and 6-class solutions were not viable.

**Table 3:** Class Enumeration Fit Indices

			Parsimony Criteria				Clustering Criteria		Both
Classes	LL	Entropy	AIC	BIC	CAIC	ssBIC	CLC	NEC	ICL-BIC
1	-3,701.01	0.00	7,442.01	7,521.84	7,541.84	7,458.38	7,402.01	1.00	7,521.84
2	-3,248.25	39.37	6,578.50	6,742.15	6,783.15	6,612.06	6,575.24	0.09	6,820.89
3	-3,175.62	77.34	6,475.24	6,722.71	6,784.71	6,525.98	6,505.92	0.15	6,877.39
4	-3,122.80	94.27	6,411.61	6,742.90	6,825.90	6,479.54	6,434.14	0.16	6,931.44

Notes: LL = Log Likelihood; AIC = Akaike Information Criterion; BIC = Bayesian Information Criterion; CAIC = Consistent Akaike Information Criterion; ssBIC = Sample Size-Adjusted Bayesian Information Criterion; CLC = Classification Likelihood Criterion; NEC = Normalized Entropy Criterion; ICL-BIC = Integrated Completed Likelihood-Bayesian Information Criterion

Latent class membership for the 4-class model suggested that 17.6% of the sample would fall into Class 1 (*Moderate ACEs/High BCEs*). About 15.3% of the sample would be classified into Class 2 (*High ACEs/Moderate BCEs*). Latent class membership suggested that 48.3% of the sample would fall into Class 3 (*Low ACEs/High BCEs*). About 18.8% of the sample would be classified into Class 4 (*Low ACEs/Moderate BCEs*). Further, classification accuracy was high in the 4-class model, with approximately 84.8% probability of being correctly classified into Class 1, with 95.1% probability of being correctly classified into Class 2, with 96.1% probability of being correctly classified into Class 3, and with 83.4% probability of being correctly classified into Class 4 (see Online Supplement Table 2 for 4-class model response probabilities).

We retained the 4-class model for further analysis for two reasons. First, when model fit is equivocal between class solutions, theory should guide class determination [69]. In this instance, Resilience Theory suggests that a degree of nuance in levels of BCEs may show mitigating influences on the link between ACEs and psychological distress [31]. Second, the 4-class solution largely replicates the only prior LCA study using item-level ACE/BCE information [33].

**Aim 2: To analyze the association of latent classes of childhood experiences with psychological distress and STBs.** For the study sample 1, the inclusion of outcomes (see Online Supplement Table 3) showed the classes were still best defined by response patterns in the 4-class model with similar posterior probability of class membership (see Online Supplement Table 4). Online Supplement Table 5 contains demographic characteristics for each class. Class comparisons showed significant differences in suicidal ideation, depression, and anxiety in a consistent pattern (see Online Supplement Table 6 for overall statistical tests). For each of these outcomes, *High ACEs/Moderate BCEs* (Class 2) had significantly higher scores than *Moderate ACEs/High BCEs* (Class 1); *Low ACEs/Moderate BCEs* (Class 4) had significantly higher scores than *Moderate ACEs/High BCEs* (Class 1); *High ACEs/Moderate BCEs* (Class 2) had significantly higher scores than *Low ACEs/High BCEs* (Class 3); and *Low ACEs/Moderate BCEs* (Class 4) had significantly higher scores than *Low ACEs/High BCEs* (Class 3; see Table 4 for descriptive statistics by class). The same pattern was consistent for post-traumatic stress, except there was no significant difference in scores for *Moderate ACEs/High BCEs* (Class 1) compared to the *High ACEs/Moderate BCEs* (Class 2). There were no other significant differences between classes on outcome measures (see Table 4 for descriptive statistics by class).

**Table 4:** Psychological distress, wellbeing, and suicidal thoughts/behaviour outcomes means and standard errors

	Moderate ACEs/High BCEs		High ACEs/Moderate BCEs		Low ACEs/High BCEs		Low ACEs/Moderate BCEs	
	Mean	*S.E.*	Mean	*S.E.*	Mean	*S.E.*	Mean	*S.E.*
Inflection Points Study Sample								
Suicidal thinking	3.20	1.41	10.80	2.14	2.32	0.75	8.36	1.11
Depression	1.24	0.27	3.15	0.34	1.21	0.15	2.85	0.20
Post-traumatic stress	5.05	0.51	6.22	0.46	3.60	0.19	5.83	0.29
Anxiety	6.91	1.17	10.03	0.95	5.38	0.48	10.66	0.71
Hate-Motivated Behaviour Study Sample								
Depression	4.13	0.57	11.44	0.53	3.48	0.27	6.46	0.29
Anxiety	9.30	0.85	15.80	0.60	7.85	0.34	13.16	0.34
Wellbeing	24.39	0.89	15.56	0.74	24.97	0.41	19.85	0.41
Defeat	16.44	1.57	48.26	1.50	12.57	0.80	29.03	1.11
External Entrapment	3.11	1.16	29.89	1.04	2.82	0.56	14.33	0.92
Internal Entrapment	1.55	0.65	16.37	0.62	1.06	0.32	5.76	0.49

Notes: S.E.=Standard Error; Classes named after class enumeration procedures, Moderate ACEs/High BCEs = Class 1; High ACEs/Moderate BCEs = Class 2; Low ACEs/High BCEs = Class 3; Low ACEs/Moderate BCEs = Class 4

For study sample 2, the inclusion of psychological distress and STB outcomes yielded a model maintaining a similar response likelihood to the 4-class enumeration model in study sample 1 (see Online Supplement Table 3). Online Supplement Table 7 contains overall statistical tests. *High ACEs/Moderate BCEs* (Class 2) showed significantly higher scores for depression, anxiety, defeat, external and internal entrapment, compared to all other classes. *Low ACEs/Moderate BCEs* (Class 4) showed significantly higher scores for depression, anxiety, defeat, external and internal entrapment when compared to *Moderate ACEs/High BCEs* (Class 1). *Low ACEs/Moderate BCEs* (Class 4) showed significantly higher scores for depression, anxiety, defeat, external and internal entrapment when compared with *Low ACEs/High BCEs* (Class 3). Class comparisons for wellbeing revealed the same significant effects as for depression, anxiety, defeat, and internal and external entrapment. However, class comparisons were in the opposite direction for wellbeing. For instance, *Low ACEs/Moderate BCEs* (Class 4) showed significantly lower scores for wellbeing when compared to *Moderate ACEs/High BCEs* (Class 1) (see Table 4 for outcome descriptive statistics by class). No significant differences between *Moderate ACEs/High BCEs* (Class 1) and *Low ACES/High BCEs* (Class 3) for the aforementioned outcomes were observed.

Odds of self-harm ideation and self-harm behaviour were significantly higher in *Moderate ACEs/High BCEs* (Class 1; self-harm ideation 61.1% *p*_*yes*_; self-harm behaviour 54.3% *p*_*yes*_) as compared to *Low ACEs/High BCEs* (Class 3; self-harm ideation 10.8% *p*_*yes*_; self-harm behaviour 3.5% *p*_*yes*_); self-harm ideation and behaviour were also higher in *Low ACEs/Moderate BCEs* (Class 4; self-harm ideation 62.9% *p*_*yes*_; self-harm behaviour 41.3% *p*_*yes*_) when compared to *Low ACEs/High BCEs* (Class 3; self-harm ideation 10.8% *p*_*yes*_; self-harm behaviour 3.5% *p*_*yes*_). Odds of both self-harm ideation and self-harm behaviour were significantly lower in *Low ACEs/Moderate BCEs* (Class 4; self-harm ideation 62.9% *p*_*yes*_; self-harm behaviour 41.3% *p*_*yes*_) when compared to *High ACEs/Moderate BCEs* (Class 2; self-harm ideation 96.2% *p*_*yes*_; self-harm behaviour 79.5% *p*_*yes*_). Further, odds of self-harm behaviour, but not self-harm ideation, were significantly lower in *Low ACEs/High BCEs* (Class 3; self-harm behaviour 3.5% *p*_*yes*_) when compared to *High ACEs/Moderate BCEs* (Class 2; self-harm behaviour 79.5% *p*_*yes*_).

## Discussion

The purpose of this study was to extend research on ACEs and BCEs by conducting an LCA among UK adults. Our aims were to (1) assess the extent to which LCA groupings replicate; and (2) describe childhood experience latent classes by psychological distress and STBs. Overall, our findings demonstrated evidence for a 4-class model and contributed to the growing literature on resiliency which suggests benevolent experiences may buffer negative outcomes in individuals who have experienced adversity.

### Hypothesis 1: a 4-class model will best fit the ACEs/BCEs data

First, we found support for a 4-class model of ACEs/BCEs subgroups: Class 1 (*Moderate ACEs/High BCEs*), Class 2 (*High ACEs/Moderate BCEs*), Class 3 (*Low ACEs/High BCEs*), and Class 4 (*Low ACEs/Moderate BCEs*). This finding is consistent with the Johnson and colleagues study which identified 4 subgroups: Class 1 (*Low ACEs/High BCEs*), Class 2 (*Moderate ACEs/High BCEs*), Class 3 (*Moderate ACEs/Low BCEs*), and Class 4 (*High ACEs/Moderate BCEs*) [33]. There was only one discrepancy between our classification of subgroups and those identified by Johnson et al. [33] study; our study identified a *Low ACEs/Moderate BCEs* subgroup whereas Johnson and colleagues identified a *Moderate ACEs/Low BCEs* subgroup. Another similarity between our findings and the Johnson et al. study was proportion of class membership [33]. In our study and the Johnson et al. [33] study, the most common class membership was found in the *Low ACEs/High BCEs* subgroup (48.3% and 49.4% respectively); whereas the least common class membership was in the *High ACEs/Mod BCEs* subgroup (15.3% and 11.2% respectively). Although Tang and colleagues conducted LCAs of the ACEs/BCEs items separately, they did find evidence for a 3-class model of ACEs and a 4-class model of BCEs [34]. Specifically, they found evidence in their sample for a *High ACEs* subgroup and a *Low ACEs* subgroup in addition to a *High BCEs* subgroup and a *Low BCEs* subgroup. Our results, combined with the evidence from the two prior LCA studies examining item-level ACEs and BCEs, demonstrate discrete subgroups of individuals exist in their endorsement of ACEs/BCEs and that these findings may replicate to a degree across samples.

### Hypothesis 2: high ACEs/Low BCEs class will be associated with higher psychological distress and STBs

Consistent with hypothesis 2, in both study samples, the class with high ACEs had significantly higher levels of psychological distress (depression, anxiety, post-traumatic stress^3^, poorer mental well-being) and STBs (suicidal ideation, self-harm ideation and behaviour, entrapment, and defeat) compared with either the low or moderate ACEs classes. Conversely, higher levels of BCEs were generally associated with lower levels of psychological distress and STBs.

**Depression**, **anxiety**,** post-traumatic stress**,** and well-being**. We found that higher ACEs were associated with greater psychological distress symptoms. Also, the two classes with high BCEs had significantly higher scores for well-being when compared with the *Low ACEs/Moderate BCEs* Class 4. This set of results is consistent with the substantial literature on ACEs with regard to psychological distress (e.g., [15, 70, 71]) and mental well-being (e.g., [72]) in adulthood. Yet less research exists on resulting psychological distress and mental well-being in the presence of both ACEs and BCEs. One study of adults found that BCEs protected against depression symptoms regardless of level of ACEs reported [31]. Although they examined a sample of Chinese adolescents, Tang et al. found that membership in an emotional abuse class (i.e., high probability of endorsing emotional abuse/neglect) versus a higher ACEs class was associated with an increased risk for depressive symptoms [34]. They posited that stress-sensitivity theory may help explain individuals’ propensity to develop depressive symptoms due to exposure to early stressful experiences. Analyzing the content of ACEs items endorsed, in addition to the level of ACEs, may be important in understanding risk for specific psychological distress. Alternatively, emerging research suggests emotion dysregulation may help explain the relationship between early childhood experiences and psychological distress; and a moderation effect (albeit small) of BCEs on ACEs and depression, anxiety, and post-traumatic stress symptoms [73]. Finally, the beneficial role of BCEs we found aligns well with earlier research that suggests BCEs may protect against later development of psychological distress [9, 15, 26]. These findings may be explained by Resilience Theory which posits direct and moderating effects between BCEs and health consequences, where BCEs may buffer against poor health outcomes [31].

**STBs.** Overall, classes with high ACEs had significantly higher levels of STBs. Also, both high BCEs classes had significantly lower levels of STBS when compared to one of the moderate BCEs classes. Prior authors have similarly identified that increasing levels of ACEs are associated with elevated risk for suicidal ideation (e.g., [32, 74]), and suicide attempts (e.g., [75, 76]). Evidence is also present to suggest membership in a high BCEs class is associated with reduced suicidal ideation [32, 34]. Importantly, previous research has not addressed the relationships of ACEs and BCEs with defeat, entrapment, or self-injurious ideation/behaviour. Potential explanatory mechanisms for observed associations between ACEs and BCEs with STBs are numerous. For instance, possible mediators between ACEs and suicide attempts in the literature include psychological distress [77, 78], as well as chronic pain and substance use [78]. Alternatively, placed in the context of the IMV model [79], the combination of ACEs and BCEs may serve a pre-motivational function, acting as part of a diathesis-stress mechanism laying the foundational risk (or lack thereof) for the development of defeat, entrapment, and eventual suicidal and/or self-injurious ideation and behaviour.

Study findings support the overall conclusions. First, four latent classes we identified largely mirrored Johnson et al.’s study [33]. Second, high levels of ACEs or BCEs were influential on psychological distress and STBs. Third, though our study did not test statistical interactions, BCEs may buffer the impact of ACEs on mental health problems when ACEs are only low or moderate, but this pattern does not hold when ACEs are high. Finally, evidence points to the possible role of ACEs and/or BCEs on the downstream development of self-harming behaviours via defeat or entrapment, although this point needs future examination.

### Implications for theory, practice, and research

There is a relative lack of theoretical development regarding childhood experiences. Our ACEs/BCEs class findings inform theory development in this area by providing additional evidence of the important intersections between ACEs and BCEs. Our findings provide indirect support for Resiliency Theory’s Compensatory Model [30, 31]. The model highlights the role compensatory factors (e.g., BCEs) serve by exerting an opposing influence on developmental outcomes, such as promoting healthy behaviours or reducing violence, compared to risks. For instance, Crandall and colleagues [31] reported that, among community-dwelling adults, the addition of BCEs to regression models reduced the effects of ACEs to non-significance for a variety of health outcomes. Crandall et al. reasoned that the addition of BCEs supported the compensatory Resiliency Theory viewpoint. Showing the import of the present investigation, in our study, participants with high BCEs experienced protection (e.g., higher well-being) even in the presence of moderate levels of ACEs, while participants with high ACEs were unable to escape their strong influence (e.g., higher odds of self-injurious behaviour). Thus, our study offers further support of the compensatory mechanism across samples of community-dwelling adults, but through an LCA analytic approach. Resiliency Theory offers a promising lens through which the intersection of ACEs and BCEs should be further considered.

In light of STB-focused results, ACEs/BCEs are additionally relevant theories of suicide. Across theories of suicide, the conjoint influence of ACEs and BCEs may serve a foundational role influencing future STB outcomes. For instance, the fluid vulnerability theory conceptualizes suicide within a diathesis-stress model that suggests suicide is nonlinear, time-bound, and influenced by prior adverse experiences [80]. Prior adverse experiences, like ACEs, may increase an individual’s predisposed vulnerability to suicide through mechanisms such as biological processes, behavioural and physical health conditions, and psycho-social development [81]. These historical risk factors tend to be static in nature and representative of between person risk differences. The IMV model [82] also purports a diathesis-stress foundation of suicide, conceptualizations pre-existing life events (e.g., ACEs and BCEs) similarly impacting formation of the pathway to suicide. In the instance of the IMV, ACEs may directly escalate risk for feelings of defeat and entrapment. On the other hand, as evidenced by the general protective role of BCEs in this study, high BCEs potentially (directly or through stress buffering) disrupts the pathway from defeat and entrapment towards suicidal ideation and attempt. Future studies should consider testing ACEs and BCEs as pre-existing and moderating factors within these theoretical models of suicide.

Present findings hold several prevention practice implications. For instance, our results highlight the critical role ACEs may play in experiencing future psychological distress and STBs. An important aspect of the present investigation is that our samples were community-dwelling as opposed to clinical in nature. Ports and colleagues argued for the importance of considering ACEs in a comprehensive, community-based approach to suicide prevention [37]. Among their recommendations was a focus on upstream prevention approaches focused on building connectedness, fostering safe and supportive environments, enhancing educational awareness of the ACEs-suicide link, and targeting shared precipitants influencing both ACEs and suicide. Ports et al. further suggest widespread use of CDC technical packages address suicide and childhood adversity prevention. We concur with these recommendations and suggest they be extended to account for the role of BCEs. Active emphasis on promoting healthy childhood experiences as part of these community-based prevention approaches may foster long-term benefits with regard to mitigating the impacts of ACEs and development of suicide risk. Mental health providers may also draw insight from present findings. For example, providers should assess ACEs as potential risk factor for psychological distress and STBs. BCEs may offer a meaningful buffer and temper the development of psychological distress and STBs. This notion is particularly salient for mental health providers, as often time spent assessing for patient strengths at the time of assessment is minimized or overlooked [83]. Leveraging BCEs through assessment and therapeutic interventions could be a beneficial way of addressing current psychological distress [32], and may inform clinical formulation and intervention selection in the treatment of STBs.

A number of research implications are informed by the present study. Our study is only the third to investigate both ACEs and BCEs, yet, similar to both prior studies [33, 34], we did not examine a clinical sample. Replication of childhood experience classes in clinical samples is necessary. Additionally, examining long-term trajectories of classes from childhood through adulthood (e.g., prospective cohort design) is a necessary next step to support prevention and intervention efforts. An obvious and important area of research need concerns how ACEs and BCEs exert their influence within theories of suicide. Namely, ACEs and BCEs should be examined within the IMV [82] as pre-motivational drivers and possible moderators. Also, we join Crandall et al. [31] in urging researchers to examine how length and timing of experiences in childhood may impact their interaction and subsequent effect on psychological distress and STBs over the life course. Finally, the mechanisms underpinning links between childhood experiences classes and psychological distress/STBs are not understood. Starting points may be examining differential forms of ACEs given evidence that different types of victimization are linked with future risk for psychological distress [70]. Emotion regulation skills may also be a mechanism worth examining in light of research showing emotion dysregulation explains the relationship between ACEs and psychological distress [73].

### Limitations

The present study possesses a number of limitations worth noting. The study was limited by non-random sampling and a cross-sectional design. Thus, results may be affected by problems such as skewed experiences of ACEs or BCEs based on sample demographics (e.g., over or under sampling subgroups at risk for ACEs), and responses to surveys affected by the design choice (e.g., current psychological distress affecting inaccurate recall of positive versus negative childhood experiences). Conclusions must therefore be tempered with respect to generalizability and causality. Our measurement choices also provide an alternative explanation to some non-significant findings. For instance, the use of single-item self-harm questions, while common, may fail to capture the full breadth of the construct. Such an approach to measurement can reduce the likelihood of detecting significant effects. A historical event also occurred in that responses in sample 1 were collected during the COVID-19 pandemic. The timing of data collection may have resulted in over-representation of psychological distress in that sample, as several studies have noted increased mental health concerns during this time period [84]. Finally, we assessed childhood experiences among adults, opening the possibility of recall bias. To overcome these limitations, future research on childhood experiences may conduct an LCA with youth and follow their psychological health and risk for STBs over time. In such a design, childhood experiences can be documented through multiple means (e.g., behavioural observation, parent/guardian report), and unique trajectories of childhood experiences better inform Resiliency and suicide-focused theories.

The present study also has several strengths worth noting. As previously mentioned, our study is the third to investigate ACEs and BCEs; and expands upon strengths-based literature by including protective factors as opposed to focusing solely on assessing risk factors for psychopathology. Further, our study leverages the advantages of an advanced statistical technique, latent class analysis, which allows patterns in the data to drive our understanding of relevant symptoms within groups of individuals. Finally, results from our study can help inform resiliency and suicide theory development by providing additional evidence of the important intersections between ACEs and BCEs in class levels of psychological distress and STBs.

## Conclusion

ACEs are well-established as a risk factor for many negative health outcomes. However, the role of BCEs is not as well understood. Our findings, coupled with prior evidence, suggest that: (a) naturally occurring subgroups of childhood experiences may replicate, and (b) BCEs may serve a compensatory role in mitigating effects of ACEs. Thus, our findings support Resiliency Theory applied to psychological distress, and highlights the importance of future research to examine ACEs and BCEs within established suicide theories. Further, our study highlights the importance of integrating BCEs within community-based and clinical responses to ACEs, psychological distress and suicide prevention. Future work should further disentangle the complex association of ACEs and BCEs through multiple methods across community and clinical samples.

### Electronic supplementary material

Below is the link to the electronic supplementary material.

Supplementary Material 1

## Data Availability

Data are available upon request with the permission of University of Strathclyde lead investigators (Rasmussen, Russell) and Ethics Review Board.

## Footnotes

1 Present analyses are completely distinct from the primary paper that is also in process. The primary paper is focused on understanding the factors that might halt the process of engaging in suicidal or self-injurious behaviour. In addition to standardized measures of protective factors, the primary paper incorporates a number of open-ended questions which probed the reasons why someone who has considered suicidal behaviours, might not translate the thoughts into behaviours.

2 Present analyses are completely distinct from the sample 2 primary paper that is also in process. The primary paper evaluates psychometric properties of a hate-motivated behaviour instrument.

3 There was no significant difference in level of post-traumatic stress symptoms for *Moderate ACEs/High BCEs* Class 1 compared to the *High ACEs/Moderate BCEs* Class 2.

## Abbreviations

ACEs: Adverse childhood experiences
BCEs: Benevolent childhood experiences
CDC: Centers for Disease Control and Prevention
GAD-7: Generalized Anxiety Disorder-7
HADS: Hospital Anxiety and Depression Scale
HMB: Hate-Motivated Behaviour
IMV: Integrated Motivational-Volitional
LCA: Latent class analysis
MNNMM: Multivariate non-normal mixture model
PCL-2: Posttraumatic Checklist-2
PIS: Participant information sheet
PHQ-2: Patient Health Questionnaire-2
SIDAS: Suicidal Ideation Attributes Scale
STBs: Suicidal thoughts and behaviours
SWEMWBS: short version Warwick-Edinburgh Mental Wellbeing Scale
UK: United Kingdom
US: United States
