# Supplementary material for: Adverse and benevolent childhood experiences among adults in the United Kingdom: a latent class analysis
Source: BMC Public Health. 2024 Jul 30;24:2052. doi: 10.1186/s12889-024-19448-z (PMC11290251; doi:10.1186/s12889-024-19448-z)
Supplement: Supplementary file 1 — Supplementary Material 1 [file 12889_2024_19448_MOESM1_ESM.docx]

**Online Supplement**

**Inflections points study 1 data preparation**. A total of 906 responses were present in the survey data (i.e., 906 people clicked to begin the survey, but did not necessarily respond to items). We cleaned and prepared survey response data in a manner consistent with best practices to ensure quality in online survey data research (e.g., [1, 2]). We adopted the following stepwise approach. First, we dropped cases with less than 75% complete data (335 cases were dropped). Second, we deleted extreme cases based on time to completion (i.e., those falling outside the 5^th^ and 95^th^ percentiles), resulting in 28 cases being dropped. Third, we deleted cases containing complete missing data from two or more consecutive questionnaires (69 cases were dropped at this step). The final resulting analyzable sample size of 488 is consistent with percentages of simulations using similar stepwise data cleaning steps [1].

**Hate-motivated behaviour study data preparation**. A total of 692 persons opened the survey link and consented to participate. We approached data cleaning in a multi-step process consistent with best practice literature in ensuring data quality in online or internet survey research (e.g., [2, 3]). First, we dropped respondents with less than 70% complete data or with complete missing data on > 2 measures of interest, resulting in 174 case deletions. Most of these deleted cases had no or very few item responses. Second, we dropped extreme cases with respect to time to completion (i.e., those lower than the 5^th^ and higher than the 95^th^ percentile) because brief or extensive time to completion can signal inattentive or other poor responding [2]. This step resulted in 54 case deletions. Finally, we adopted a stringent approach to preventing duplicate responding [3] by dropping cases of repetitive IP addresses, resulting in 16 additional case deletions. The final analyzable sample size was 447, a sample proportion consistent with simulation research on data cleaning best practices [1].

References

| 1. | Arevalo M, Brownstein NC, Whiting J, Meade CD, Gwede CK, Vadaparampil ST, et al. Strategies and lessons learned during cleaning of data from research panel participants: cross-sectional web-based health behavior survey study. JMIR Form Res. 2022;6(6):e35797. |
| --- | --- |
|  |  |
| 2. | Leiner DJ. Too fast, too straight, too weird: non-reactive indicators for meaningless data in internet surveys. Survey Research Methods. 2019;13(3):229–48. |
|  |  |
| 3. | Aust F, Diedenhofen B, Ullrich S, Musch J. Seriousness checks are useful to improve data validity in online research. Behav Res Methods. 2013;45(2):527–35. |
|  |  |

Table 1. 2-Class Response Probabilities

|  |  | Probability of "Yes" | |
| --- | --- | --- | --- |
|  |  | Class 1 | Class 2 |
| ACEs |  |  |  |
| 1 | Emotional abuse | 8.6% | 70.2% |
| 2 | Physical abuse | 4.3% | 48.8% |
| 3 | Sexual abuse | 2.3% | 18.8% |
| 4 | Emotional neglect | 6.8% | 68.0% |
| 5 | Physical neglect | 0.0% | 20.4% |
| 6 | Separation from or loss of a parent | 22.6% | 54.8% |
| 7 | Witnessed mother abused | 1.5% | 22.7% |
| 8 | Household substance abuse | 14.9% | 37.2% |
| 9 | Household mental illness | 27.0% | 63.2% |
| 10 | Household member went to prison | 1.0% | 9.2% |
| BCEs |  |  |  |
| 11 | At least one safe caregiver | 99.1% | 76.0% |
| 12 | At least one good friend | 99.1% | 85.0% |
| 13 | Beliefs that gave comfort | 71.4% | 30.1% |
| 14 | Liked school | 76.0% | 40.1% |
| 15 | At least one teacher who cared | 93.3% | 59.8% |
| 16 | Had good neighbours | 85.5% | 50.0% |
| 17 | Non-caregiver for support/advice | 91.8% | 45.8% |
| 18 | Opportunities for fun | 100.0% | 86.4% |
| 19 | Like/feel comfortable with self | 66.6% | 25.5% |
| 20 | Predictable home routines | 94.4% | 54.0% |

Notes: ACEs=Adverse Childhood Experiences; BCEs=Benevolent Childhood Experiences

Table 2. 4-Class Response Probabilities

|  |  | Probability of "Yes" | | | |
| --- | --- | --- | --- | --- | --- |
|  |  | Class 1 | Class 2 | Class 3 | Class 4 |
| ACEs |  |  |  |  |  |
| 1 | Emotional abuse | 65.3% | 100.0% | 0.6% | 10.6% |
| 2 | Physical abuse | 36.3% | 65.6% | 1.6% | 9.9% |
| 3 | Sexual abuse | 18.8% | 25.5% | 0.0% | 2.9% |
| 4 | Emotional neglect | 35.2% | 85.6% | 2.6% | 34.6% |
| 5 | Physical neglect | 4.9% | 31.8% | 0.0% | 5.5% |
| 6 | Separation from or loss of a parent | 54.7% | 63.6% | 16.7% | 31.3% |
| 7 | Witnessed mother abused | 18.4% | 30.1% | 0.8% | 1.6% |
| 8 | Household substance abuse | 49.0% | 35.6% | 5.6% | 29.2% |
| 9 | Household mental illness | 63.1% | 65.4% | 20.2% | 43.5% |
| 10 | Household member went to prison | 4.9% | 14.5% | 0.0% | 3.6% |
| BCEs |  |  |  |  |  |
| 11 | At least one safe caregiver | 95.6% | 59.4% | 99.0% | 94.4% |
| 12 | At least one good friend | 100.0% | 78.8% | 100.0% | 87.7% |
| 13 | Beliefs that gave comfort | 76.3% | 22.1% | 77.2% | 19.4% |
| 14 | Liked school | 81.0% | 27.0% | 81.0% | 35.0% |
| 15 | At least one teacher who cared | 90.7% | 54.8% | 97.0% | 58.8% |
| 16 | Had good neighbours | 83.5% | 29.8% | 88.8% | 61.6% |
| 17 | Non-caregiver for support/advice | 84.4% | 38.1% | 95.8% | 51.6% |
| 18 | Opportunities for fun | 96.2% | 76.6% | 100.0% | 98.6% |
| 19 | Like/feel comfortable with self | 66.3% | 12.7% | 72.0% | 24.2% |
| 20 | Predictable home routines | 80.0% | 34.6% | 98.4% | 74.8% |

Notes: ACEs=Adverse Childhood Experiences; BCEs=Benevolent Childhood Experiences

Table 3. Response Probabilities Across Classification Models

|  | Probability of "Yes" | | | | | | | | | | | |
| --- | --- | --- | --- | --- | --- | --- | --- | --- | --- | --- | --- | --- |
|  | LCA Model | | | | IPS Distal Outcomes Model | | | | HMBC Distal Outcomes Model | | | |
|  | Class 1 | Class 2 | Class 3 | Class 4 | Class 1 | Class 2 | Class 3 | Class 4 | Class 1 | Class 2 | Class 3 | Class 4 |
| ACEs |  |  |  |  |  |  |  |  |  |  |  |  |
| 1 | 65.3% | 100.0% | 0.6% | 10.6% | 27.3% | 100.0% | 4.9% | 24.4% | 63.1% | 100.0% | 4.4% | 22.9% |
| 2 | 36.3% | 65.6% | 1.6% | 9.9% | 17.8% | 68.4% | 1.4% | 5.3% | 34.9% | 73.9% | 0.0% | 10.4% |
| 3 | 18.8% | 25.5% | 0.0% | 2.9% | 8.3% | 20.3% | 2.9% | 6.4% | 15.6% | 33.0% | 0.0% | 4.0% |
| 4 | 35.2% | 85.6% | 2.6% | 34.6% | 12.6% | 71.1% | 1.4% | 30.4% | 61.9% | 88.9% | 3.3% | 19.5% |
| 5 | 4.9% | 31.8% | 0.0% | 5.5% | 4.2% | 18.2% | 0.0% | 0.0% | 18.8% | 32.4% | 0.0% | 0.0% |
| 6 | 54.7% | 63.6% | 16.7% | 31.3% | 80.1% | 52.1% | 3.7% | 29.8% | 84.1% | 68.6% | 29.5% | 26.6% |
| 7 | 18.4% | 30.1% | 0.8% | 1.6% | 29.0% | 22.8% | 0.0% | 1.4% | 46.9% | 28.0% | 2.1% | 2.0% |
| 8 | 49.0% | 35.6% | 5.6% | 29.2% | 46.3% | 41.6% | 4.9% | 16.7% | 95.3% | 53.1% | 6.4% | 6.1% |
| 9 | 63.1% | 65.4% | 20.2% | 43.5% | 73.6% | 52.2% | 0.1% | 37.4% | 94.9% | 63.2% | 16.2% | 46.3% |
| 10 | 4.9% | 14.5% | 0.0% | 3.6% | 17.9% | 3.1% | 1.8% | 1.6% | 20.3% | 0.0% | 1.0% | 1.0% |
| BCEs |  |  |  |  |  |  |  |  |  |  |  |  |
| 11 | 95.6% | 59.4% | 99.0% | 94.4% | 95.8% | 66.2% | 100.0% | 100.0% | 91.9% | 63.4% | 100.0% | 98.0% |
| 12 | 100.0% | 78.8% | 100.0% | 87.7% | 93.9% | 76.3% | 100.0% | 94.1% | 100.0% | 82.1% | 100.0% | 95.1% |
| 13 | 76.3% | 22.1% | 77.2% | 19.4% | 88.3% | 34.2% | 71.6% | 42.3% | 54.7% | 20.1% | 84.5% | 60.9% |
| 14 | 81.0% | 27.0% | 81.0% | 35.0% | 83.1% | 44.5% | 96.0% | 45.4% | 69.4% | 23.7% | 95.0% | 55.0% |
| 15 | 90.7% | 54.8% | 97.0% | 58.8% | 72.9% | 53.1% | 96.9% | 76.2% | 92.1% | 57.7% | 100.0% | 84.6% |
| 16 | 83.5% | 29.8% | 88.8% | 61.6% | 77.3% | 39.9% | 89.7% | 61.3% | 66.4% | 43.9% | 93.1% | 72.3% |
| 17 | 84.4% | 38.1% | 95.8% | 51.6% | 95.4% | 39.9% | 93.8% | 69.8% | 85.7% | 46.6% | 100.0% | 80.2% |
| 18 | 96.2% | 76.6% | 100.0% | 98.6% | 100.0% | 92.2% | 100.0% | 98.9% | 100.0% | 81.2% | 100.0% | 99.0% |
| 19 | 66.3% | 12.7% | 72.0% | 24.2% | 100.0% | 24.6% | 81.7% | 19.9% | 60.9% | 8.9% | 97.4% | 38.2% |
| 20 | 80.0% | 34.6% | 98.4% | 74.8% | 96.5% | 57.7% | 98.3% | 78.4% | 62.2% | 40.9% | 100.0% | 91.7% |

Notes: LCA=Latent Class Analysis; IPS=Inflection Points Study; HMBC=Hate-Motivated Behaviour Checklist; ACES=Adverse Childhood Experiences; BCEs=Benevolent Childhood Experiences; ACEs & BCEs item numbers are consistent with those in Tables 1 & 2; Classes named after class enumeration procedures correspond to Moderate ACEs/High BCEs (Class 1), High ACEs/Moderate BCEs (Class 2), Low ACEs/High BCEs (Class 3), and Low ACEs/Moderate BCEs (Class 4).

Table 4. Posterior Probability of Class Membership Across Models

|  | Posterior Probability | | |
| --- | --- | --- | --- |
| Class | LCA Model | IPS Model | HMBC Model |
| 1 | 17.6% | 8.8% | 14.8% |
| 2 | 15.3% | 14.2% | 10.0% |
| 3 | 48.3% | 42.8% | 36.8% |
| 4 | 18.8% | 34.1% | 38.4% |

Notes: Posterior probabilities of classification shown for LCA model sample (*n* = 400), Inflection Points Study model (IPS Model) with distal outcomes (*n* = 218), and Hate-Motivated Behaviour Study model (HMBC Model) with distal outcomes (*n* = 182).

Table 5. Demographic Characteristics by Class Enumeration

| Demographic | Class 1 | Class 2 | Class 3 | Class 4 |
| --- | --- | --- | --- | --- |
|  | *M* (*SD*) | *M* (*SD*) | *M* (*SD*) | *M* (*SD*) |
| Age | 29 (13) | 26 (7) | 27 (12) | 28 (10) |
|  |  |  |  |  |
|  | *n* (%) | *n* (%) | *n* (%) | *n* (%) |
| Gender |  |  |  |  |
| Man | 12 (17.6%) | 17 (27.4%) | 35 (17.7%) | 16 (22.2%) |
| Woman | 55 (80.9%) | 44 (71.0%) | 162 (81.8%) | 55 (76.4%) |
| Non-binary (genderqueer, gender fluid) | 0 (0.0%) | 1 (1.6%) | 1 (0.5%) | 0 (0.0%) |
| Agender | 1 (1.5%) | 0 (0.0%) | 0 (0.0%) | 1 (1.4%) |
| Race |  |  |  |  |
| White | 62 (91.2%) | 59 (95.2%) | 185 (93.4%) | 67 (93.1%) |
| Racial Minority | 6 (8.8%) | 3 (4.8%) | 13 (6.6%) | 5 (6.9%) |
| Sexual Orientation |  |  |  |  |
| Heterosexual | 30 (44.1%) | 25 (40.3%) | 95 (48.0%) | 39 (54.2%) |
| Gay | 3 (4.4%) | 4 (6.5%) | 2 (1.0%) | 3 (4.2%) |
| Lesbian | 24 (35.3%) | 15 (24.2%) | 81 (40.9%) | 16 (22.2%) |
| Bisexual | 8 (11.8%) | 12 (19.4%) | 10 (5.1%) | 9 (12.5%) |
| Pansexual | 2 (2.9%) | 3 (4.8%) | 1 (0.5%) | 3 (4.2%) |
| Queer | 0 (0.0%) | 0 (0.0%) | 1 (0.5%) | 1 (1.4%) |
| Asexual | 1 (1.5%) | 1 (1.6%) | 3 (1.5%) | 0 (0.0%) |
| Other or multiple identities | 0 (0.0%) | 2 (3.2%) | 5 (2.5%) | 1 (1.4%) |
| **Total** | **68** | **62** | **198** | **72** |

Notes: N=400; M=Mean; SD=Standard deviation. Assignments are based on the most likely class membership. Classes named after class enumeration procedures correspond to Moderate ACEs/High BCEs (Class 1), High ACEs/Moderate BCEs (Class 2), Low ACEs/High BCEs (Class 3), and Low ACEs/Moderate BCEs (Class 4).

Table 6. Class Comparisons on Psychological Distress and Suicidal Ideation Outcomes: Inflection Points Study Sample

|  |  | χ^2^ | *p* |
| --- | --- | --- | --- |
| Suicidal thinking |  |  |  |
|  | Moderate ACES/High BCEs vs. High ACEs/Moderate BCEs | **8.61** | **.003** |
|  | Moderate ACES/High BCEs vs. Low ACEs/High BCEs | 0.29 | .587 |
|  | Moderate ACES/High BCEs vs. Low ACEs/Moderate BCEs | **7.85** | **.005** |
|  | High ACEs/Moderate BCEs vs. Low ACEs/High BCEs | **14.13** | **< .001** |
|  | High ACEs/Moderate BCEs vs. Low ACEs/Moderate BCEs | 0.93 | .335 |
|  | Low ACEs/High BCEs vs. Low ACEs/Moderate BCEs | **17.91** | **< .001** |
| Depression |  |  |  |
|  | Moderate ACES/High BCEs vs. High ACEs/Moderate BCEs | **18.55** | **< .001** |
|  | Moderate ACES/High BCEs vs. Low ACEs/High BCEs | 0.01 | .908 |
|  | Moderate ACES/High BCEs vs. Low ACEs/Moderate BCEs | **21.49** | **< .001** |
|  | High ACEs/Moderate BCEs vs. Low ACEs/High BCEs | **27.40** | **< .001** |
|  | High ACEs/Moderate BCEs vs. Low ACEs/Moderate BCEs | 0.53 | .466 |
|  | Low ACEs/High BCEs vs. Low ACEs/Moderate BCEs | **38.57** | **< .001** |
| Post-traumatic stress |  |  |  |
|  | Moderate ACES/High BCEs vs. High ACEs/Moderate BCEs | 2.79 | .095 |
|  | Moderate ACES/High BCEs vs. Low ACEs/High BCEs | **6.79** | **.009** |
|  | Moderate ACES/High BCEs vs. Low ACEs/Moderate BCEs | 1.69 | .194 |
|  | High ACEs/Moderate BCEs vs. Low ACEs/High BCEs | **27.64** | **< .001** |
|  | High ACEs/Moderate BCEs vs. Low ACEs/Moderate BCEs | 0.55 | .499 |
|  | Low ACEs/High BCEs vs. Low ACEs/Moderate BCEs | **35.97** | **< .001** |
| Anxiety |  |  |  |
|  | Moderate ACES/High BCEs vs. High ACEs/Moderate BCEs | **4.15** | **.042** |
|  | Moderate ACES/High BCEs vs. Low ACEs/High BCEs | 1.41 | .235 |
|  | Moderate ACES/High BCEs vs. Low ACEs/Moderate BCEs | **7.21** | **.007** |
|  | High ACEs/Moderate BCEs vs. Low ACEs/High BCEs | **19.21** | **< .001** |
|  | High ACEs/Moderate BCEs vs. Low ACEs/Moderate BCEs | 0.25 | .615 |
|  | Low ACEs/High BCEs vs. Low ACEs/Moderate BCEs | **33.42** | **< .001** |

Notes: Suicidal thinking=Suicidal Ideation Attributes Scale; Depression=Patient Health Questionnaire-2; Post-traumatic stress=Posttraumatic Checklist-2; Anxiety=Generalized Anxiety Disorder-7; S.E.=Standard Error; Moderate ACES/High BCEs=Class 1; High ACES/Moderate BCES=Class 2; Low ACEs/High BCES=Class 3; Low ACES/Moderate BCEs=Class 4

Table 7. Class Comparisons on Psychological Distress and Suicidal Thoughts and Behaviour Outcomes: Hate-Motivated Behaviour Study Sample

|  |  | χ2 | *OR* | *p* |
| --- | --- | --- | --- | --- |
| Depression |  |  |  |  |
|  | Moderate ACES/High BCEs vs. High ACEs/Moderate BCEs | **95.83** | *** | **<.001** |
|  | Moderate ACES/High BCEs vs. Low ACEs/High BCEs | 1.07 | *** | .301 |
|  | Moderate ACES/High BCEs vs. Low ACEs/Moderate BCEs | **14.25** | *** | **<.001** |
|  | High ACEs/Moderate BCEs vs. Low ACEs/High BCEs | **180.25** | *** | **<.001** |
|  | High ACEs/Moderate BCEs vs. Low ACEs/Moderate BCEs | **75.01** | *** | **<.001** |
|  | Low ACEs/High BCEs vs. Low ACEs/Moderate BCEs | **57.41** | *** | **<.001** |
| Anxiety |  |  |  |  |
|  | Moderate ACES/High BCEs vs. High ACEs/Moderate BCEs | **40.52** | *** | **<.001** |
|  | Moderate ACES/High BCEs vs. Low ACEs/High BCEs | 2.68 | *** | .102 |
|  | Moderate ACES/High BCEs vs. Low ACEs/Moderate BCEs | **18.63** | *** | **<.001** |
|  | High ACEs/Moderate BCEs vs. Low ACEs/High BCEs | **133.36** | *** | **<.001** |
|  | High ACEs/Moderate BCEs vs. Low ACEs/Moderate BCEs | **14.62** | *** | **<.001** |
|  | Low ACEs/High BCEs vs. Low ACEs/Moderate BCEs | **128.11** | *** | **<.001** |
| Wellbeing |  |  |  |  |
|  | Moderate ACES/High BCEs vs. High ACEs/Moderate BCEs | **61.29** | *** | **<.001** |
|  | Moderate ACES/High BCEs vs. Low ACEs/High BCEs | 0.35 | *** | .555 |
|  | Moderate ACES/High BCEs vs. Low ACEs/Moderate BCEs | **24.44** | *** | **<.001** |
|  | High ACEs/Moderate BCEs vs. Low ACEs/High BCEs | **124.90** | *** | **<.001** |
|  | High ACEs/Moderate BCEs vs. Low ACEs/Moderate BCEs | **26.45** | *** | **<.001** |
|  | Low ACEs/High BCEs vs. Low ACEs/Moderate BCEs | **81.61** | *** | **<.001** |
| Defeat |  |  |  |  |
|  | Moderate ACES/High BCEs vs. High ACEs/Moderate BCEs | **228.02** | *** | **<.001** |
|  | Moderate ACES/High BCEs vs. Low ACEs/High BCEs | 4.99 | *** | .026 |
|  | Moderate ACES/High BCEs vs. Low ACEs/Moderate BCEs | **54.81** | *** | **<.001** |
|  | High ACEs/Moderate BCEs vs. Low ACEs/High BCEs | **456.56** | *** | **<.001** |
|  | High ACEs/Moderate BCEs vs. Low ACEs/Moderate BCEs | **128.31** | *** | **<.001** |
|  | Low ACEs/High BCEs vs. Low ACEs/Moderate BCEs | **171.12** | *** | **<.001** |
| External Entrapment |  |  |  |  |
|  | Moderate ACES/High BCEs vs. High ACEs/Moderate BCEs | **303.01** | *** | **<.001** |
|  | Moderate ACES/High BCEs vs. Low ACEs/High BCEs | 0.05 | *** | .819 |
|  | Moderate ACES/High BCEs vs. Low ACEs/Moderate BCEs | **89.45** | *** | **<.001** |
|  | High ACEs/Moderate BCEs vs. Low ACEs/High BCEs | **529.38** | *** | **<.001** |
|  | High ACEs/Moderate BCEs vs. Low ACEs/Moderate BCEs | **134.08** | *** | **<.001** |
|  | Low ACEs/High BCEs vs. Low ACEs/Moderate BCEs | **130.06** | *** | **<.001** |
| Internal Entrapment |  |  |  |  |
|  | Moderate ACES/High BCEs vs. High ACEs/Moderate BCEs | **270.35** | *** | **<.001** |
|  | Moderate ACES/High BCEs vs. Low ACEs/High BCEs | 0.45 | *** | .501 |
|  | Moderate ACES/High BCEs vs. Low ACEs/Moderate BCEs | **33.72** | *** | **<.001** |
|  | High ACEs/Moderate BCEs vs. Low ACEs/High BCEs | **473.43** | *** | **<.001** |
|  | High ACEs/Moderate BCEs vs. Low ACEs/Moderate BCEs | **181.19** | *** | **<.001** |
|  | Low ACEs/High BCEs vs. Low ACEs/Moderate BCEs | **67.85** | *** | **<.001** |
| Self-Harm Ideation |  |  |  |  |
|  | Moderate ACES/High BCEs vs. High ACEs/Moderate BCEs | *** | 0.06 | *ns* |
|  | Moderate ACES/High BCEs vs. Low ACEs/High BCEs | *** | **12.99** | **< .05** |
|  | Moderate ACES/High BCEs vs. Low ACEs/Moderate BCEs | *** | 0.93 | *ns* |
|  | Low ACEs/High BCEs vs. High ACEs/Moderate BCEs | *** | 0.01 | *ns* |
|  | Low ACEs/Moderate BCEs vs. High ACEs/Moderate BCEs | *** | **0.07** | **< .05** |
|  | Low ACEs/Moderate BCEs vs. Low ACEs/High BCEs | *** | **14.03** | **< .05** |
| Self-Harm Behaviour |  |  |  |  |
|  | Moderate ACES/High BCEs vs. High ACEs/Moderate BCEs | *** | 0.31 | *ns* |
|  | Moderate ACES/High BCEs vs. Low ACEs/High BCEs | *** | **32.78** | **< .05** |
|  | Moderate ACES/High BCEs vs. Low ACEs/Moderate BCEs | *** | 1.69 | *ns* |
|  | Low ACEs/High BCEs vs. High ACEs/Moderate BCEs | *** | **0.01** | **< .05** |
|  | Low ACEs/Moderate BCEs vs. High ACEs/Moderate BCEs | *** | **0.18** | **< .05** |
|  | Low ACEs/Moderate BCEs vs. Low ACEs/High BCEs | *** | **19.40** | **< .05** |

Notes: OR=Odds Ratio; class comparison order shifts for Self-Harm Ideation and Self-Harm Behaviour.
